# Address to the Section of Dental and Oral Surgery

**Published:** 1890-09

**Authors:** Jacob L. Williams

**Affiliations:** Boston, Mass., Chairman of the Section


### Address to the Section of Dental and Oral Surgery.

BY JACOB L. WILLIAMS, M.D., OF BOSTON, MASS., CHAIRMAN OF THE SECTION.

Delivered at the Forty-First Annual Meeting of the American Medical Association at Nashville, Tenn., May 20, 1890.

On a brief review of recent advancements in matters pertaining to this section, we may surely say that there has been very substantial progress during the past year.

Mention should be made of laborious and successful researches on the origin and correction of oral deformities, which have added much to our supply of positive knowledge.

The study of antiseptics has been zealously pursued, further demonstrating their properties, not only as antiseptic in various degrees, but also their qualities as irritant or non-irritant, and as coagulating or non-coagulating.

Attention has been given, and knowledge has been gained in regard to the effects of naso-pharyngeal growths; on the form of the mouth, and the perversion of its functions, often to the injury of general health.

The study of special histology has given us positive evidence of some things previously suspected to exist, and pending researches promise new discoveries.

The science of chemistry has continued to lend its aid. And the esthetics of practice have had increased attention given to them. In regard to professional ethics it may be said that there is still room for further advancement.

But of greatest and most fundamental importance, I think, we may regard the increasing devotion to the consideration and study of the laws of health and disease, on which must be based all intelligent practice in any branch of the healing art.

In a field devoted to agriculture there may be various departments, some for grains, some for fruits, some for flowers; but all must be governed by the laws of vegetable growth and thrift.

A disregard of those laws, though accompanied by the most elaborate or ingenious agricultural implements, used by most skilful hands, will surely not result in the largest degree of success. So with the healing art; whoever is best prepared with knowledge of the laws of life, health, and disease, and their relations to professional practice, will find his trained skill the most successful.

It is to be hoped that, in the colleges and schools, increased time and attention will be given to the study of these fundamental principles.

It should not be forgotten that the healing art, though in some of its branches bringing mechanical skill and chemical knowledge to its aid, is itself the beneficent purpose or substance of practice; the design of surgery not being for mechanical display, nor of medicine for chemical demonstrations.

Hippocrates said, "In no one thing does mortal man more resemble the immortal gods than in giving health to man." And so far as mechanics and chemistry are made therapeutic and prophylactic aids in securing health to mankind, in so far do they become ennobled beyond their ordinary application to "inanimate things."

In earlier years there were only a few qualified practitioners who devoted their knowledge and skill to the treatment of the whole oral cavity, while the larger number gave their attention simply to the teeth; and so the specialty was called "dentistry."

But at this day, when knowledge of the principles of medicine and surgery is more general, and more commonly made available in the treatment of the whole oral cavity, and with reference to its influence on the health of the system, the old term seems too limited. And what now would be a more proper and comprehensive name for the specialty so practiced, than the word *Oristry*?

It is gratifying to know of an increasing general sentiment or principle pervading this as well as general practice, which perhaps can not be better expressed than in the line of our poet Holmes, when he wrote, as the motto of our profession, "Our duty is to save."[1]

1 "The Two Armies," by Dr. O. W. Holmes.

Every practitioner knows that he is bound in honor to *do his best* for the patient, and the laws of the land hold him to that duty.

If a case for special practice comes to him, and a competent specialist is available, he naturally feels that he is doing his best by referring the patient to the specialist. But if such special treatment is unattainable, he is bound in honor and by the common law to do all he can to save life, or limb, or member of the patient.

In the variety of legislation in different States, two at least, recognize this obligation in their statutes. But what shall we say of some State enactments that contravene the great purpose of all humane and proper practice? and assume to prohibit a physician from doing any thing in a specialty except to *destroy* a member or organ of the human body; for doing which, also, he, as well as the specialist, may become legally liable for damages.

Special statutes can not properly prohibit a physician or surgeon from *doing his best* for the benefit of a patient whatever may be the ailment.

We have the satisfaction of knowing that this section will be ably represented at the International Congress at Berlin by several of its members of well-known erudition.

---

Vegetarian Diet.—Dujardin-Beaumetz (*Wiener klin. Wochenschrift*, April 10, 1890) claims that this diet thoroughly subserves alimentation of the organism; the best proof of which is furnished by the poor peasants who do not eat meat, yet are strong and healthy. This diet is of therapeutic importance in certain diseases. A vegetable diet limits to a minimum the production of toxines, such as neurin, muscarin, etc. It is indicated in insufficient functional activity of the kidneys and alimentary canal, indeed, in all similar conditions where an accumulation of ptomaines in the blood might prove dangerous. It is also indicated in "putrid diarrhœa." In diseases of the stomach a vegetable diet is especially indicated as the intestines are principally employed in its digestion, thus affording the stomach considerable rest. In the uric acid diatheses this diet is also recommended.—*Occidental Med. Times.*

